# Supplementary figures and images for: Genome-wide analysis of 3′-untranslated regions supports the existence of post-transcriptional regulons controlling gene expression in trypanosomes
Source: PeerJ. 2013 Jul 30;1:e118. doi: 10.7717/peerj.118 (PMC3728762; doi:10.7717/peerj.118)

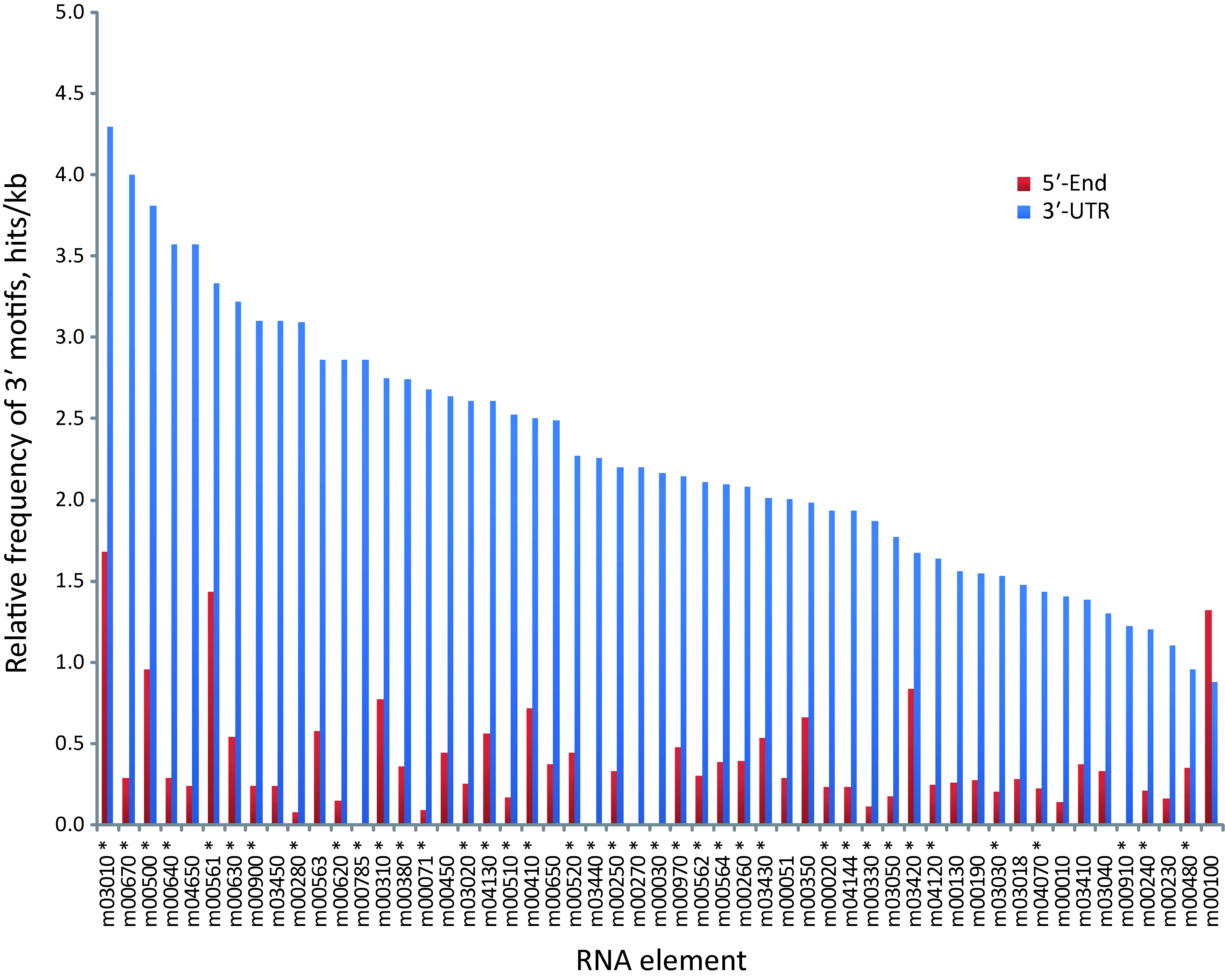

Supplement: Figure S1 — Frequency of motifs (total hits/kb) in KEGG groups fractioned in two subsets composed of 5′-end and 3′-UTR. Searches were performed using the elements identified in the 3′-UTR as queries against the estimated 5′-end of transcripts (see Methods). *, statistically significant RNA motifs from Table 2. [file peerj-01-118-s001.jpg]

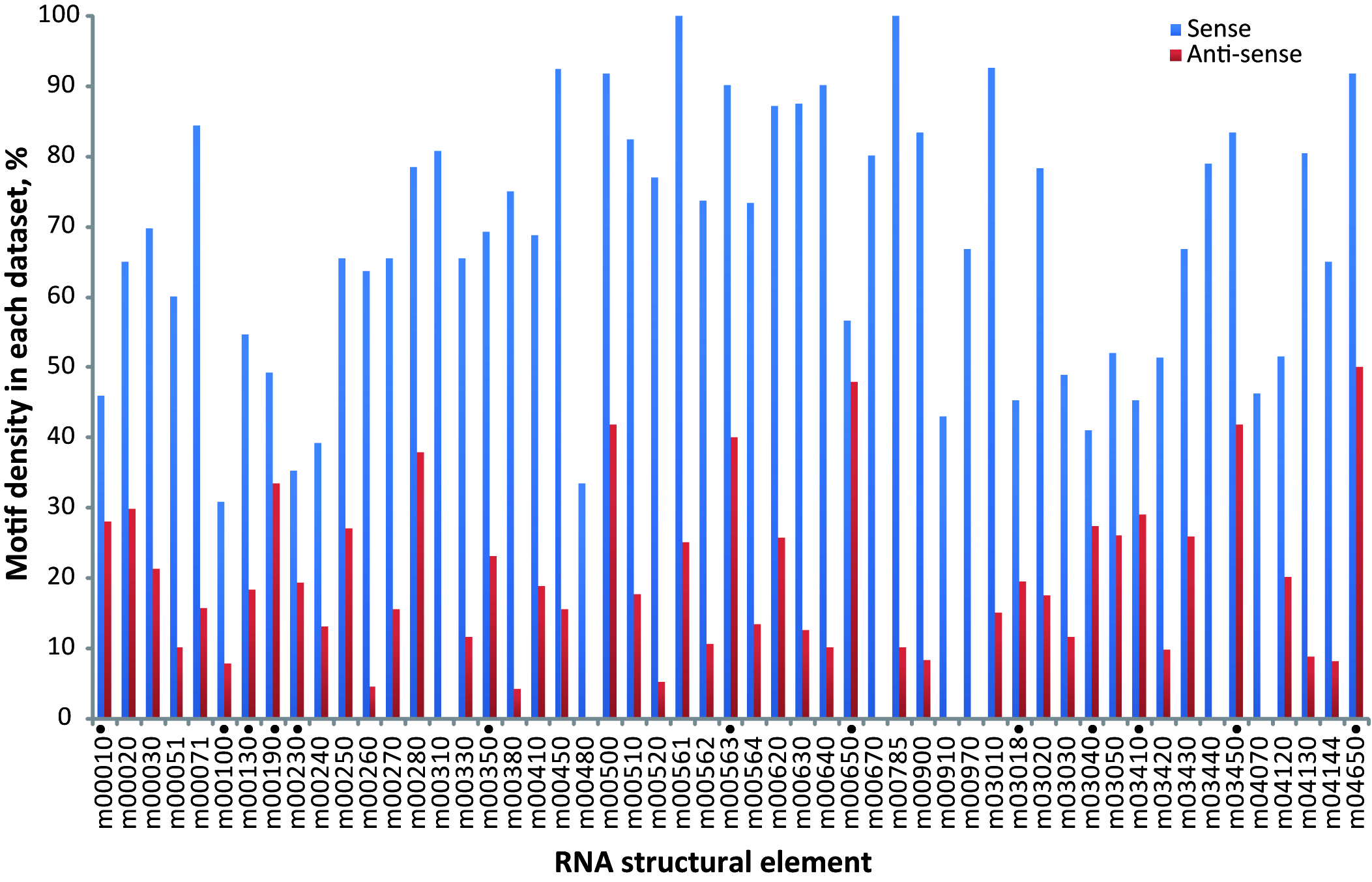

Supplement: Figure S2 — Percentage of motifs located in sense (blue) and anti-sense (red) orientation in the 3′-UTR. RNA elements marked with a black circle have no statistical significance (χ2 test, FDR 5%). [file peerj-01-118-s002.jpg]

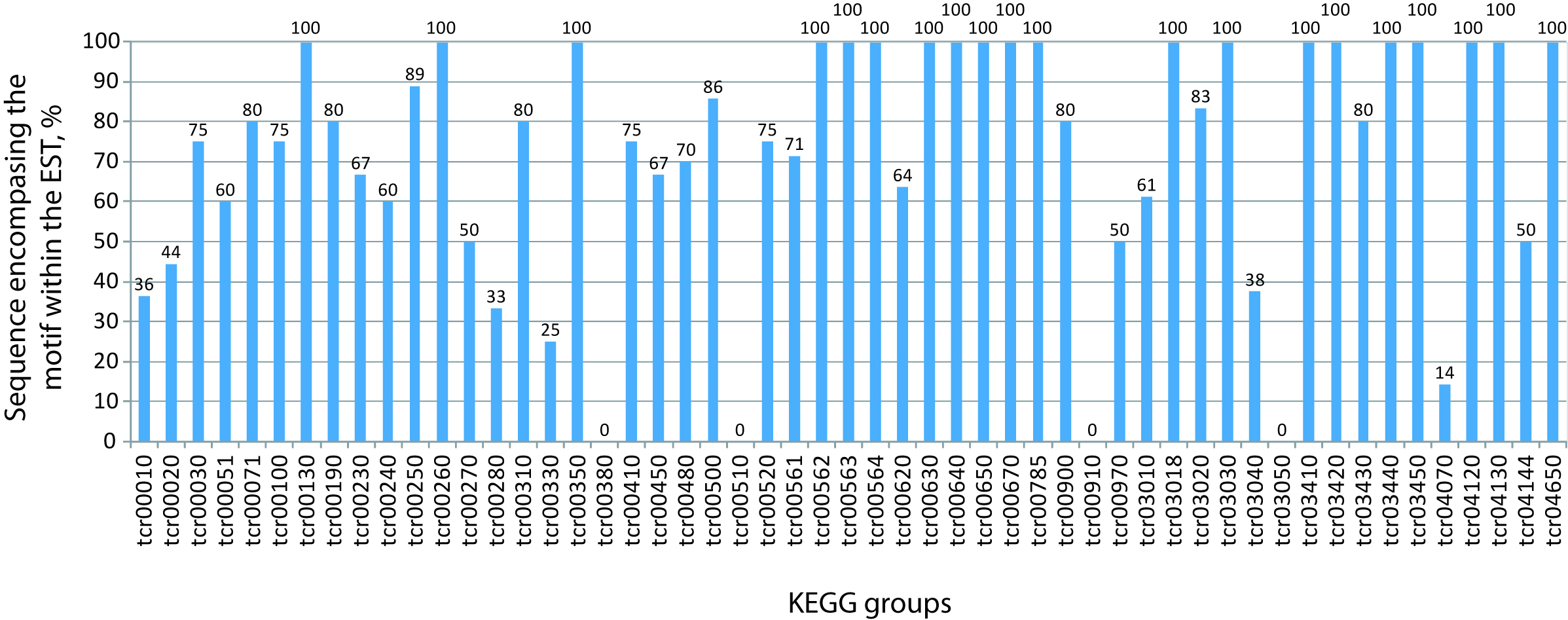

Supplement: Figure S3 — Chart of the percentage of sequences harboring the motif within the EST hit in each of the 53 KEGG groups analyzed here. Overall, 43 out of 53 categories have at least 50% of the BLAST sequences containing the complete motif within the EST hit, reinforcing the idea that our datasets could be used to identify putative regulatory RNA elements. [file peerj-01-118-s003.jpg]

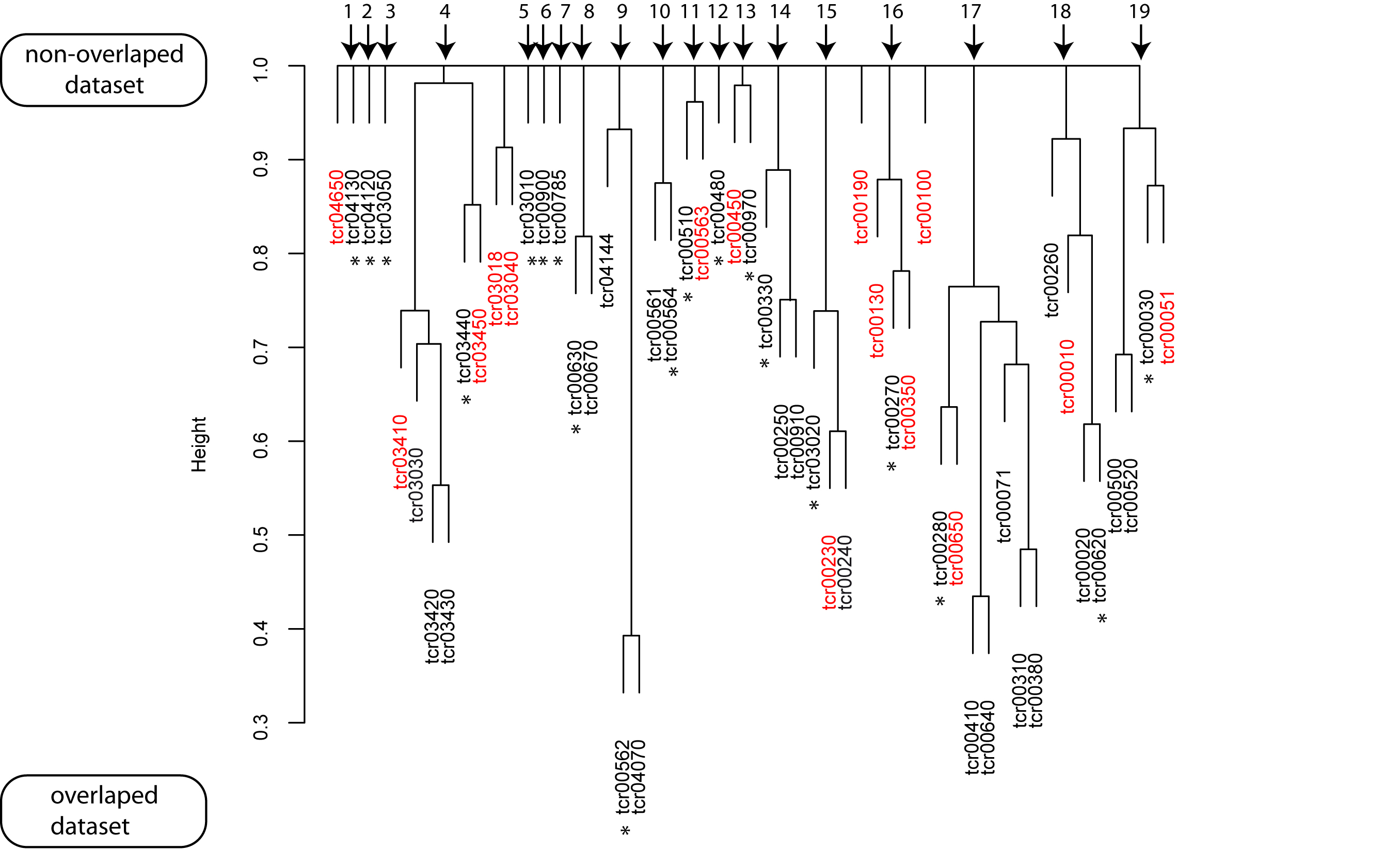

Supplement: Figure S4 — The input metabolic dataset, 53 KEGG groups, were hierarchically clustered into 23 different branches that do not share any gene with any other branch. The schematic representation shows a graphic with a height scale that varies between 0 (completely overlapped sets) to 1 (totally different sets). Next, we selected 19 groups with non-overlapped genes which contain significant conserved RNA motifs (marked with asterisks) to perform a comprehensive analysis of RNA motif representation (see Fig. 5). [file peerj-01-118-s004.jpg]
