# Supplementary material for: Genome-wide analysis of 3′-untranslated regions supports the existence of post-transcriptional regulons controlling gene expression in trypanosomes
Source: PeerJ. 2013 Jul 30;1:e118. doi: 10.7717/peerj.118 (PMC3728762; doi:10.7717/peerj.118)
Supplement: File S2 — Sequence alignments of the 53 cis-elements obtained for each set of sequences were used to plot motif logos using WebLogo version 2.8.2 (2005-09-08) (http://weblogo.berkeley.edu/) (Crooks et al. 2004). Five elements contain U-rich sequences (m00260, m00500, m00900, m03010 and m04144), 14 have AU-rich sequences (m00010, m00020, m00190, m00230, m00250, m00270, m00520, m03018, m03030, m03040, m03050, m03410, m03420 and m04650) and three are enriched in GU nts (m00051, m00970 and m03020). Nonetheless, most of the structured elements did not show a preferential enrichment at the primary sequence level and have a variable nucleotide composition. [file peerj-01-118-s006.pdf]

Supplemental File 2. List of the 53 Motifs identified in the complete KEGG dataset used in this work. Sequence logo, RNA secondary structure (in bracket notation) and consensus nucleotide sequence are shown for each element.

RNA motif: **m00010**

Seq. logo:

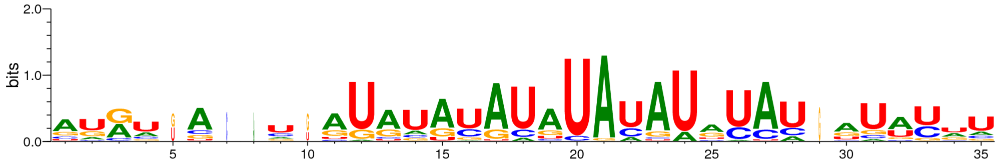

Struct.: ::<<<<<<<<<<<____>>>>>>>>>>>::
Consensus: auGuacaUauAuaUAUAuaUauAuguaCuu

RNA motif: **m00020**

Seq. logo:

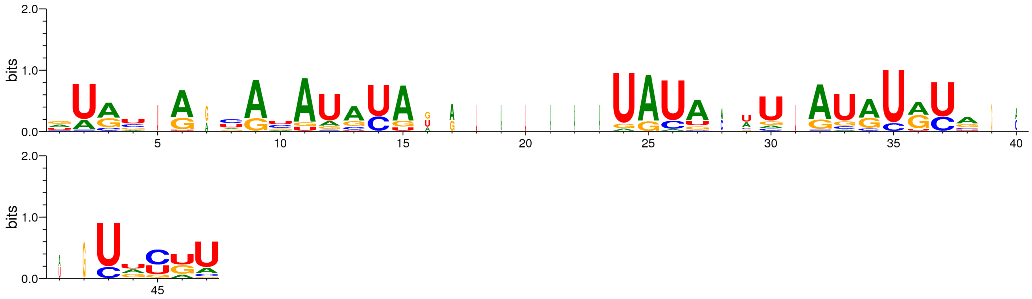

Struct.: ::<<<<<<<<<<<____>>>>>>>>>>>::
Consensus: gUGcaCacAuauaUAUAuauaUguGugCgU

RNA motif: **m00030**

Seq. logo:

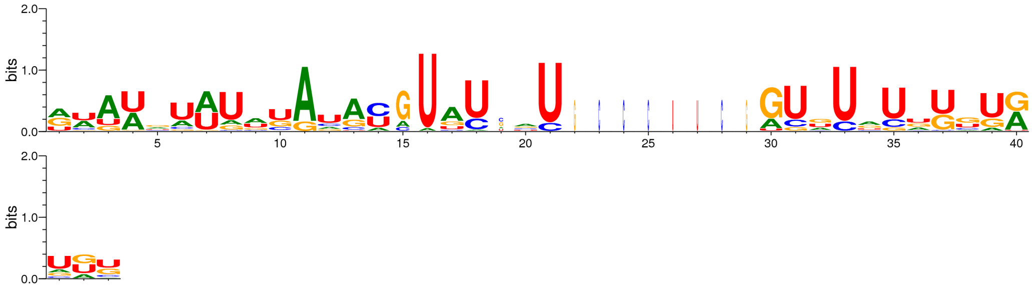

Struct.: :::::::<<<<<<<______>>>>>>>:::::::
Consensus: guAugUauagAcaCGUaUaUGugUcugUgUGUgu

RNA motif: **m00051**

Seq. logo:

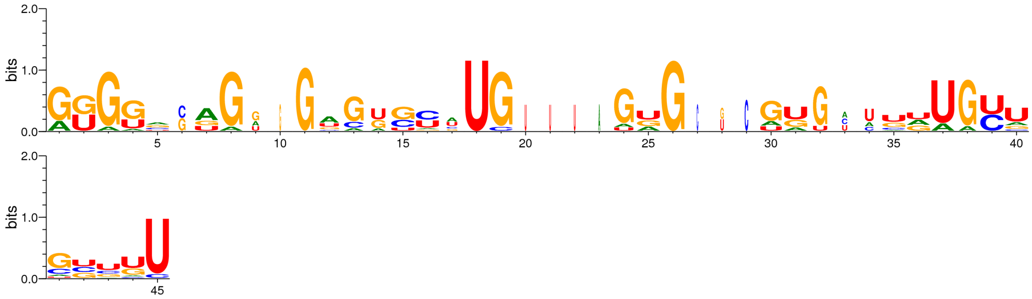

Struct.: :::<<<<<<<-<<_____>>->>>->>>>:::
Consensus: GuGgCAGGccacCUGGgGGgGggUGCUGcccU

RNA motif: **m00071**

Seq. logo:

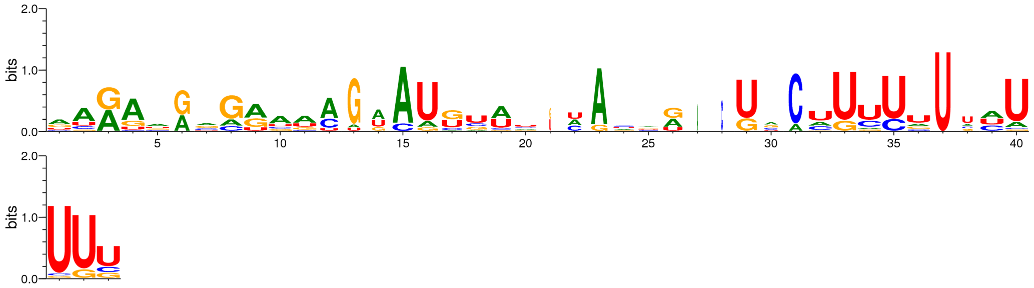


```
Struct.:    :::&lt;&lt;-&lt;&lt;&lt;&lt;&lt;---&lt;&lt;&lt;______&gt;&gt;&gt;--&gt;&gt;&gt;&gt;&gt;-&gt;&gt;:::
Consensus:  aAAagGcggagAGaAuguAccAacgUcCcuccgUcuUUu
```

RNA motif: **m00100**

Seq. logo:

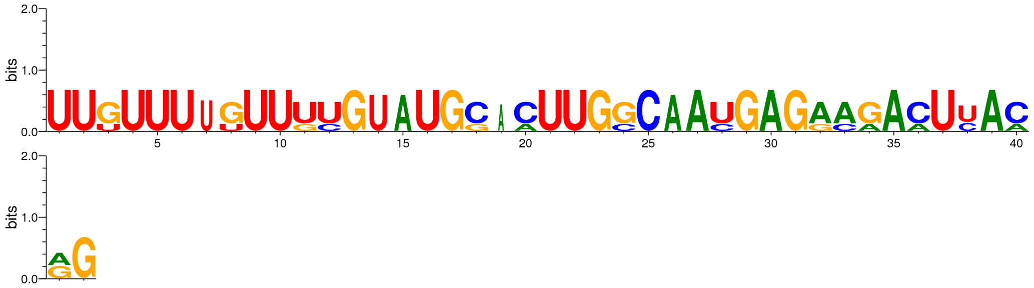


```
Struct.:    :&lt;&lt;&lt;---&lt;&lt;&lt;&lt;&lt;---&lt;&lt;&lt;____&gt;&gt;&gt;-----&gt;&gt;&gt;&gt;&gt;--&gt;&gt;&gt;:
Consensus:  UuGUUUUGUugcGUAuGCCUUGGCaAUGAGgcgACUuACgG
```

RNA motif: **m00130**

Seq. logo:

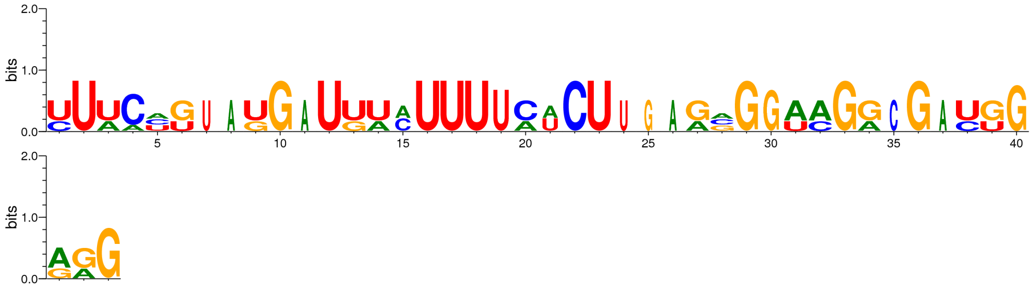


```
Struct.:    :&lt;&lt;&lt;&lt;&lt;---&lt;&lt;_____________&gt;&gt;---&gt;&gt;&gt;&gt;&gt;:
Consensus:  cuuCCguGUGuCUUUUCaCUGCGGaCGGGuGGagG
```

RNA motif: **m00190**

Seq. logo:

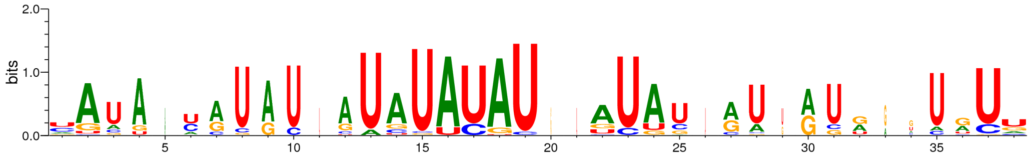


```
Struct.:    ::&lt;&lt;&lt;&lt;&lt;&lt;&lt;&lt;&lt;&lt;______&gt;&gt;&gt;&gt;&gt;&gt;&gt;&gt;&gt;&gt;::
Consensus:  CAuAcauAuAUAUAUAUAUAUaUaugUaUu
```

RNA motif: **m00230**

Seq. logo:

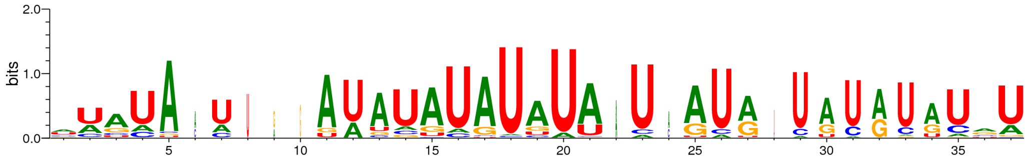


```
Struct.:    ::::&lt;&lt;&lt;&lt;&lt;&lt;&lt;&lt;&lt;&lt;____&gt;&gt;&gt;&gt;&gt;&gt;&gt;&gt;&gt;&gt;::::
Consensus:  auAUAuAuAUAUAUAUAUAUAUAUaUaUAUaU
```

RNA motif: **m00240**

Seq. logo:

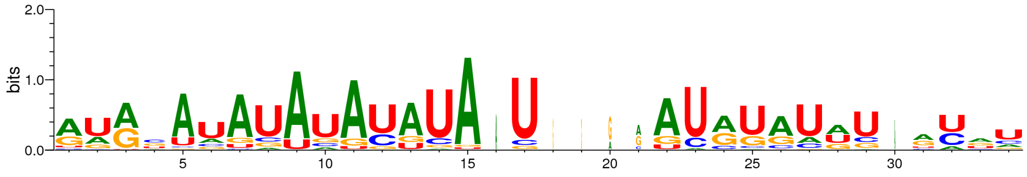

```
Struct.:     :<<-<<<<<<<<____>>>>>>>>->>:
Consensus:   AgGCAuAuauAuAUAUaUauaUaUgCcu
```

RNA motif: **m00250**

Seq. logo:

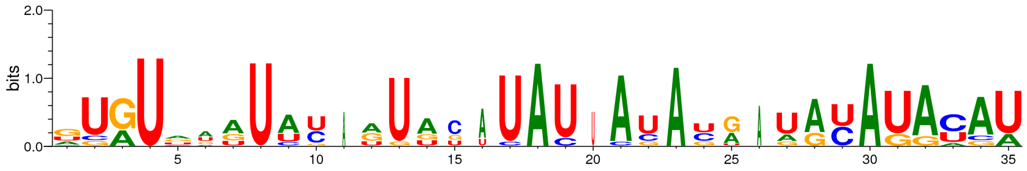

```
Struct.:     :<<<<-<<<<<<<_____>>>>>>>->>>>:
Consensus:   guGuaaguaCgUaCUAUAuAcGuacAuaCaU
```

RNA motif: **m00260**

Seq. logo:

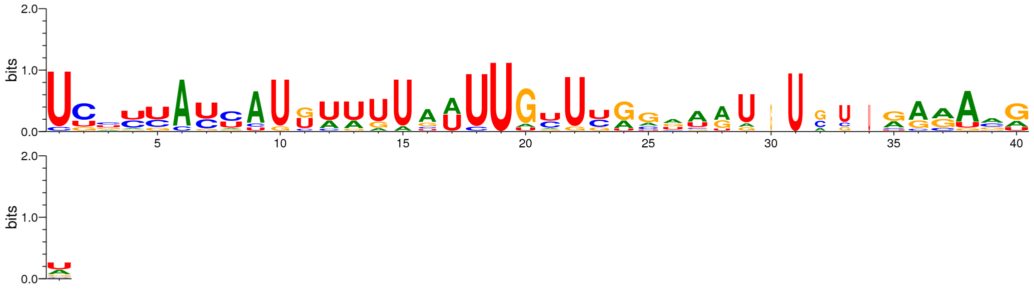

```
Struct.:     :<<<<-<<---<<<<<______>>>>>----->>>>>>:
Consensus:   UCguCAcCAUgacguauUUGuuugcguAUUCuGgGacGa
```

RNA motif: **m00270**

Seq. logo:

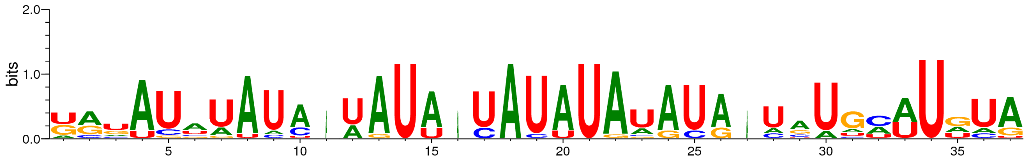

```
Struct.:     ::::::<<<<<<<________>>>>>>>::::::
Consensus:   uagAUauAuauaUAUAUAUAuAuauaUgCaUgUA
```

RNA motif: **m00280**

Seq. logo:

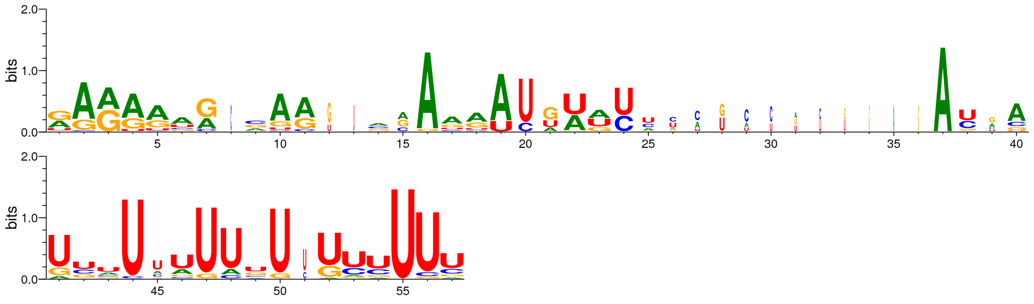

```
Struct.:     :<<<<<<<<<<-<<<<_________>>>>>>>>>>>>>>:
Consensus:   gAgggagcaaagAgGaUguaUcACAuCcUuuuguuccuUu
```

RNA motif: **m00310**

Seq. logo:

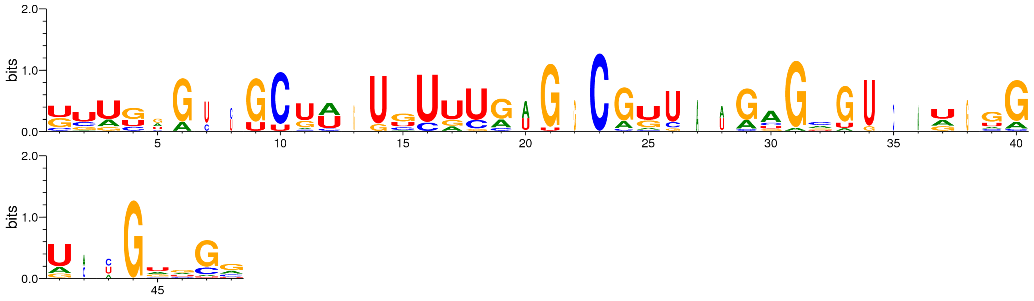

Struct.: :<<<-<<<<<<<<<______>>>>>->>>>->>>:
Consensus: gggcGGCuaUGuucGGCGuUgagCGUugGUGgccG

RNA motif: **m00330**

Seq. logo:

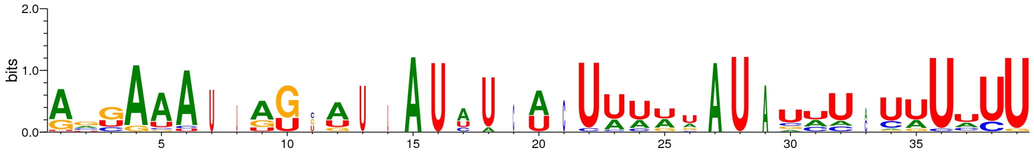

Struct.: ::<<<-<<<<<<______>>>>>>->>>::
Consensus: AccAaAagaAUcUAUuuagAUucuUuUcUU

RNA motif: **m00350**

Seq. logo:

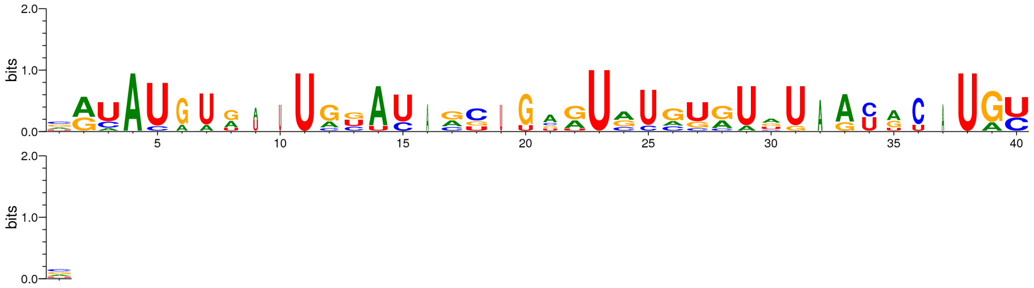

Struct.: <<<<-<<<<<<<<<<______>>>>>>>>>>>>>>
Consensus: gGuAUGuGugCaCgCGaGUaUGuGuGuaCaCUgCc

RNA motif: **m00380**

Seq. logo:

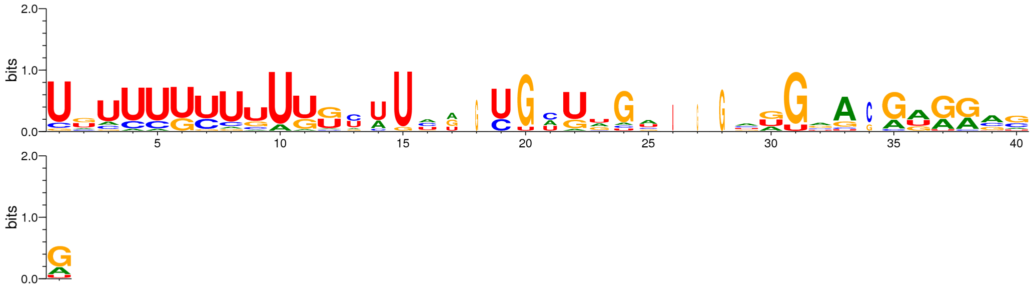

Struct.: :<<<<<<<<<<<-<<<____>>>>>>>>>>>>>>:
Consensus: UGucuuCUcUggCcUCUGCUGGgcuGgAGaggaCG

RNA motif: **m00410**

Seq. logo:

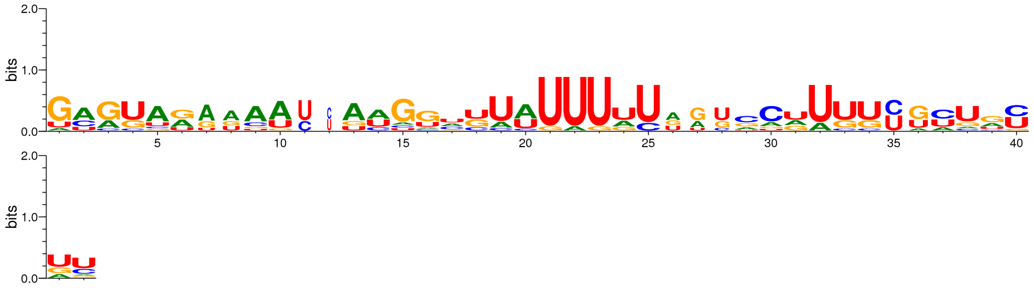

Struct.: <<<<<<--<<-<<<<<<<______>>>>>>>>>-->>>>>>

Consensus: gaGcaGAAaAuAcGGaccAUUUuUgguCCgUUuCgCugCuc

RNA motif: **m00450**

Seq. logo:

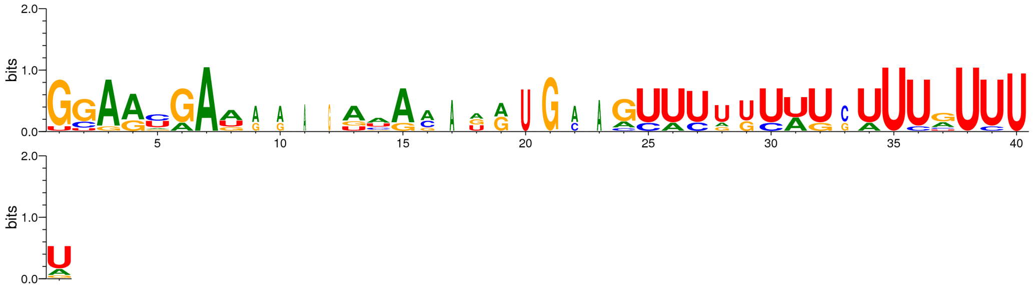

Struct.: ::<<<<<<<<<<<______>>>>>>>>>>>::

Consensus: GGAacGAaaaacggGGUUUcguuuuUUguUUu

RNA motif: **m00480**

Seq. logo:

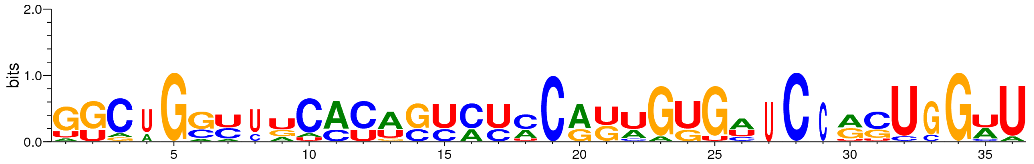

Struct.: ::<<<<<<<<<<<____>>>>>>>->>>>:::

Consensus: GGCGGCuCCCaCCCuCCGGuGGGaCGCUGGaU

RNA motif: **m00500**

Seq. logo:

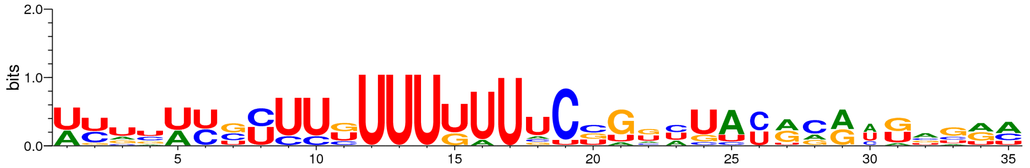

Struct.: :<<<<<<<__________________>>>->>>>:

Consensus: aCgCuCGCUucUUUgUUuCCGccUACGCGagGcGC

RNA motif: **m00510**

Seq. logo:

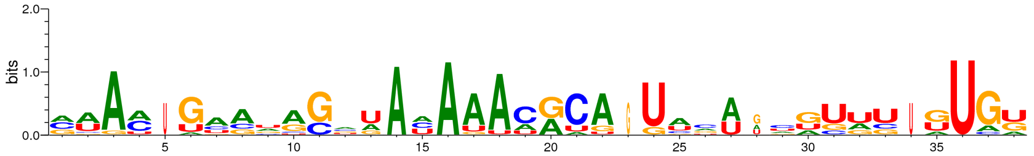

Struct.: ::<<<<<<--<<<________>>>-->>>>>>::

Consensus: caACgcauAGcuAcAAACGCAUagAcgugcGUGu

RNA motif: **m00520**

Seq. logo:

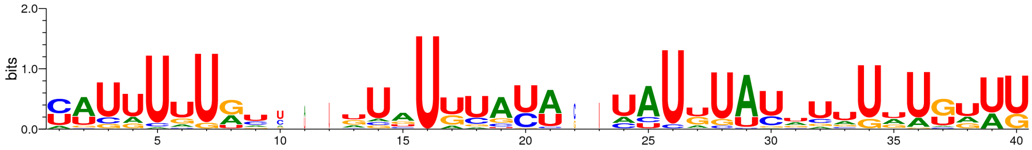

Struct.: :::::::::::::::::::::::::::::::::::
Consensus: CAuuUuUGcgUaUuuAcAuAUgUAUuuuUaUGuuu

RNA motif: **m00561**

Seq. logo:

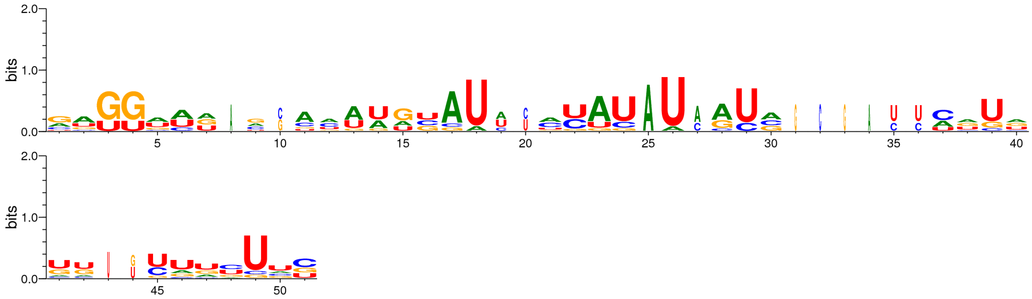

Struct.: <<<<<<<<<<<<<<<<_______>>>>>>>>>>>>>>>>
Consensus: GGgGgagcccagGGAucCuAuAUaUCCcugggcucCuCC

RNA motif: **m00562**

Seq. logo:

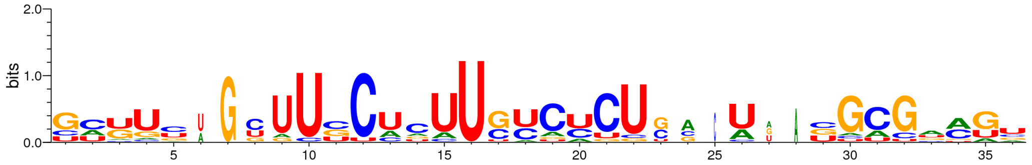

Struct.: <<<<<<<__________________>>>>>>>
Consensus: gCGuCGcuUCCuCUUGuCuCUGaucgCGaCGc

RNA motif: **m00563**

Seq. logo:

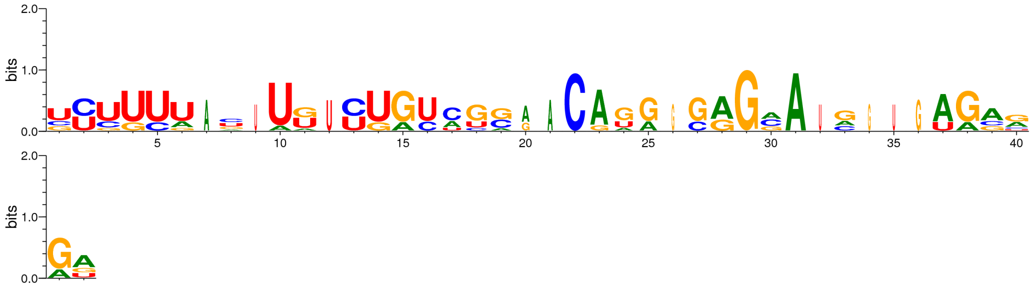

Struct.: <<<<<<<<<<--<<____>>-->>>>>>>>>>
Consensus: cCcgcuCUGCuGCCgGCAGGGAGCAGagcgGg

RNA motif: **m00564**

Seq. logo:

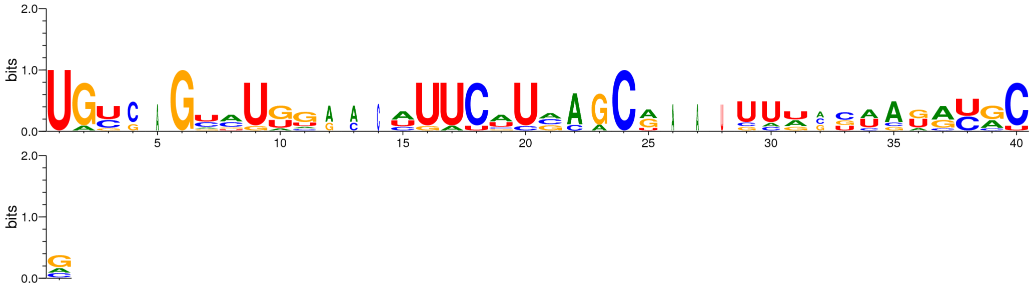

Struct.: :<<<<<<<<<____________>>>>>>>>>:
Consensus: UGCGCCUuGcUUCAUGAGCAuUgCaAGGCGCG

RNA motif: **m00620**

Seq. logo:

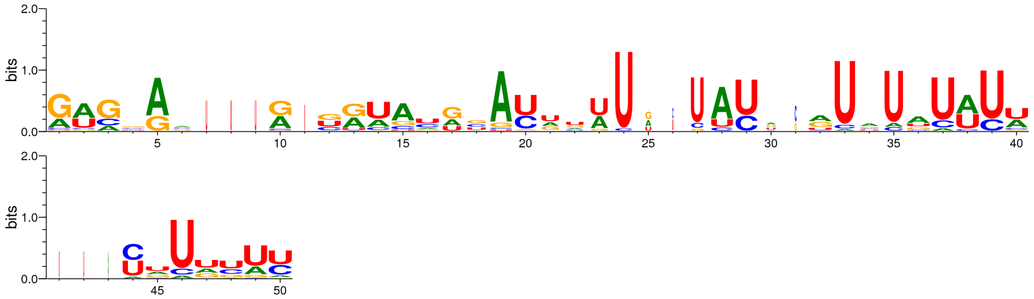

Struct.: :<<<<<<<<<<<<<<_________>>>>>>>>>>>>>>:
Consensus: GaGcacGgguacgcACuuuUUAUgUacguacuCgugCuC

RNA motif: **m00630**

Seq. logo:

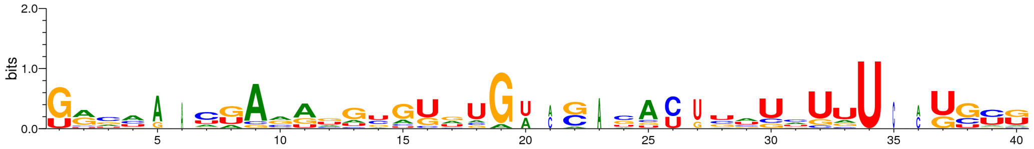

Struct.: :<<<-<<<<<<-<<<___>>>>>>>>>->>>:
Consensus: GgCCCcaGagGuGuGuGCCaCCcuCugUGGcc

RNA motif: **m00640**

Seq. logo:

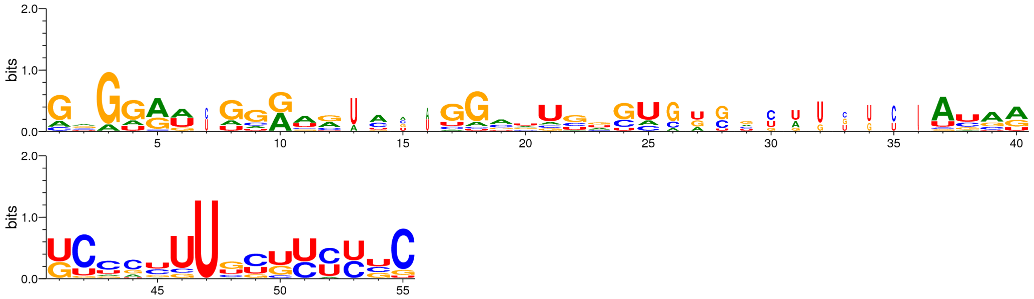

Struct.: <<<<<<<<<<<-<<<<<______>>>>>->>>>>>>>>>>
Consensus: GGGGGgGggaGcGGaauggGuAcaauCCCCuucCcCCCCC

RNA motif: **m00650**

Seq. logo:

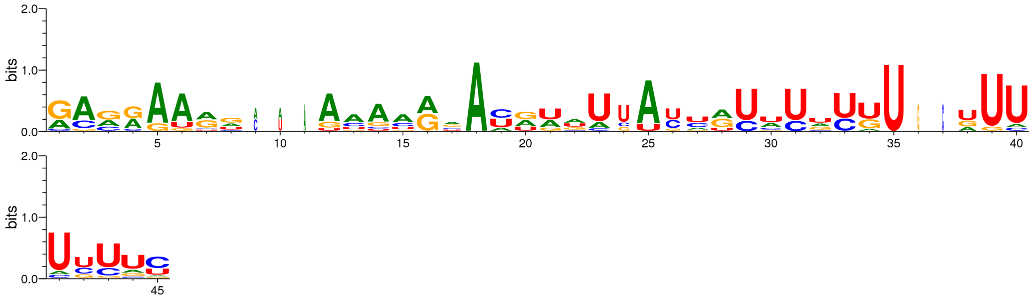

Struct.: <<<<<<<<<<<<<<<<<______>>>>>>>>>>>>>>>>>
Consensus: GAcGAaggAcaaGGAuguuUUAUcaUCCuugUcuuUCgUC

RNA motif: **m00670**

Seq. logo:

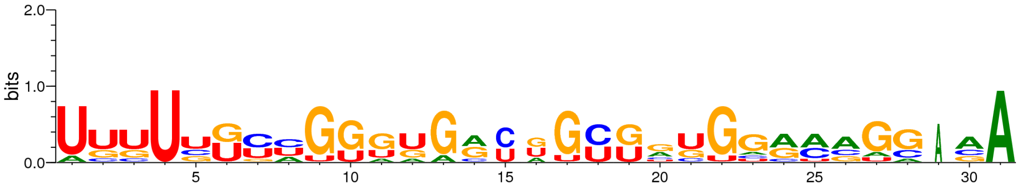

Struct.: :<<<<<<<-<<<______>>>->>>>>>>:
Consensus: UuuuuuccGgcuGACuGCgguGggaaggaA

RNA motif: **m00785**

Seq. logo:

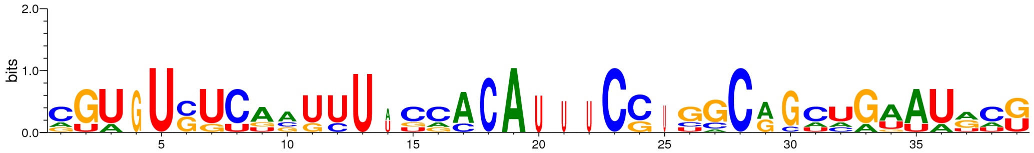

Struct.: <<<--<<<----<<_____>>---->>>-->>>
Consensus: CGuUCuCaaUUUCCACACCGGCgGCuGaAUaCG

RNA motif: **m00900**

Seq. logo:

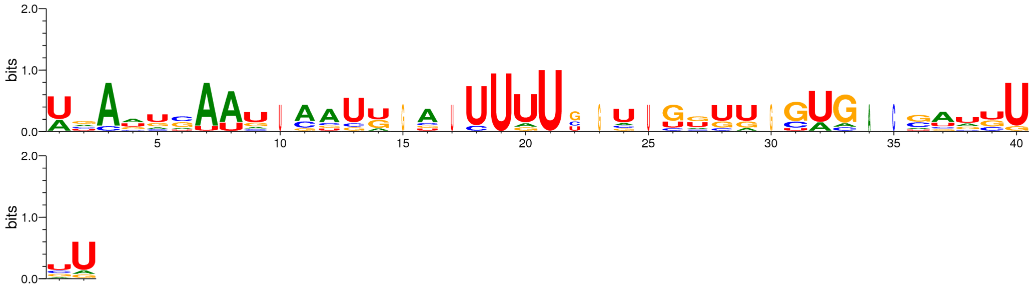

Struct.: ::::<<-<<<<<<<_____>>>>>>>->>:::::
Consensus: acAacGAAuaaugaUUUUcucguugUGCgucUcU

RNA motif: **m00910**

Seq. logo:

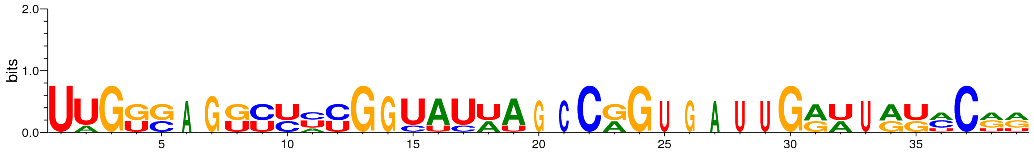

Struct.: :<<<<-<<<<<<______>>>>>>-->>>>:
Consensus: UuGGCGgCCCCGGUAuaACGGGGuUgGCCag

RNA motif: **m00970**

Seq. logo:

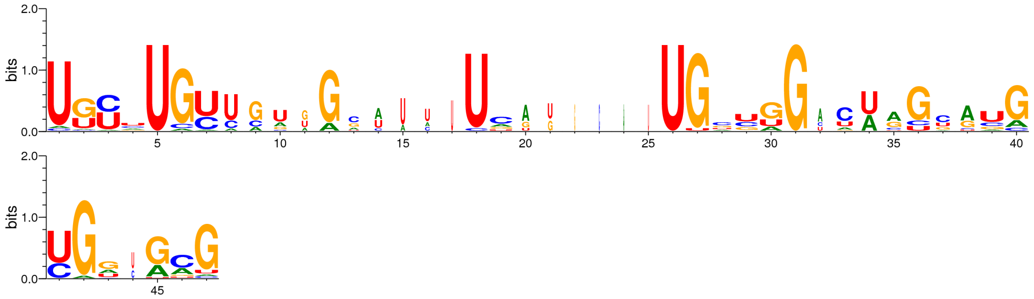

Struct.: :<<<<_______________________>>>>:

Consensus: UGCCUGUUGUGUCUGCuGGCaaGCAcGUGGGCG

RNA motif: **m03010**

Seq. logo:

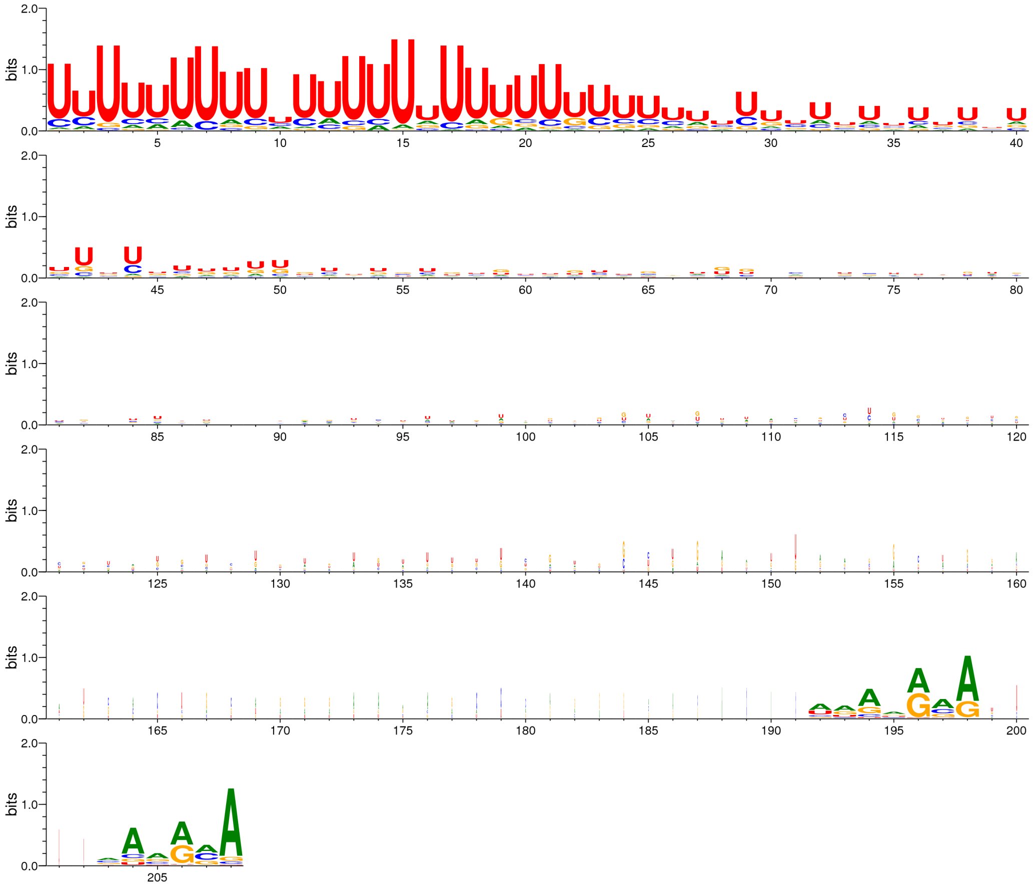

Struct.: :::
::::::::::::::::::::::

Consensus: UuUuuUUUUcUuUUUuUUuUUuuuuuucuucuuuuuuuguuugugugguugcggccgcgcggccg
aagggcagcAAAAAAAaAAAAA

RNA motif: **m03018**

Seq. logo:

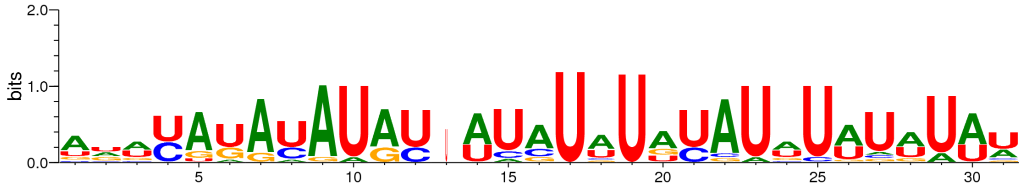

Struct.: ::::<<<<<<<<______>>>>>>>>::::

Consensus: agaUAuAuAUAuAUAUAUaUAUaUaUAUAu

RNA motif: **m03020**

Seq. logo:

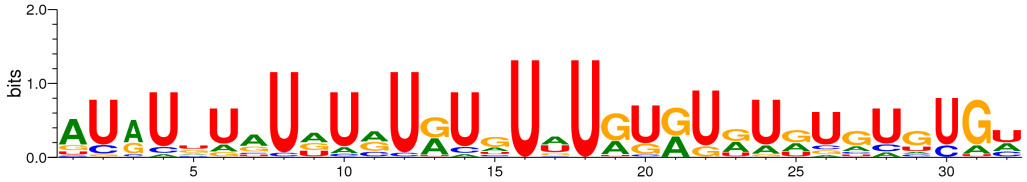

Struct.: ::::::<<<<<<________>>>>>>::::::
Consensus: AuAUguauauauGUGUAUGuauguauGUGUGa

RNA motif: **m03030**

Seq. logo:

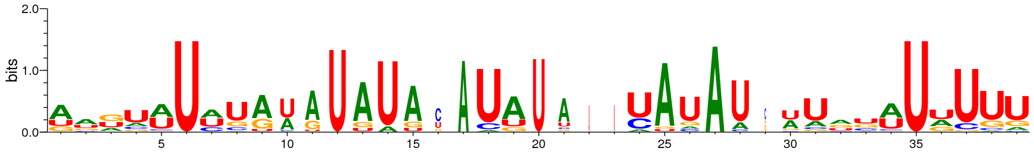

Struct.: :::::::<<<<<<<_______>>>>>>>:::::::
Consensus: aaguaUauAuAUAUAAUAUAUAUAUaUgcAUgUuu

RNA motif: **m03040**

Seq. logo:

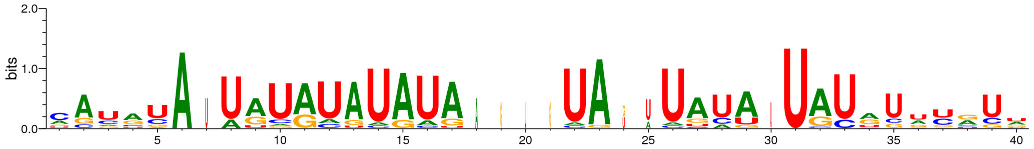

Struct.: :<<<--<<<<<<<<____>>>>>>>>-->>>:
Consensus: CacauAuAUAUAUAUAUAUAUAUAUauuugua

RNA motif: **m03050**

Seq. logo:

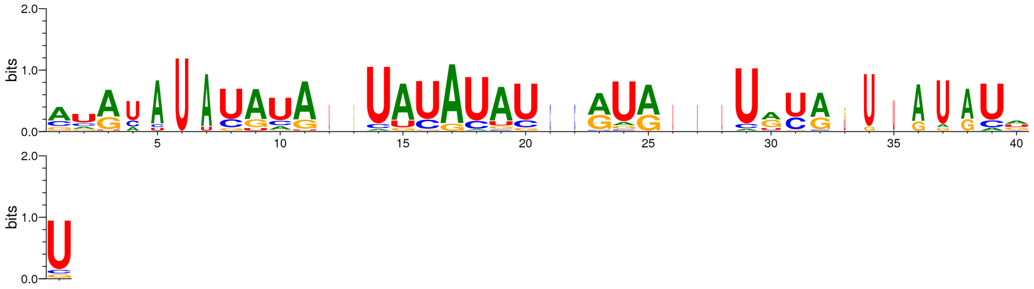

Struct.: ::<<<<<<<<<<<<____>>>>>>>>>>>>::
Consensus: AuauAuAuacAuAuAUAUaUaUguaUaUauaU

RNA motif: **m03410**

Seq. logo:

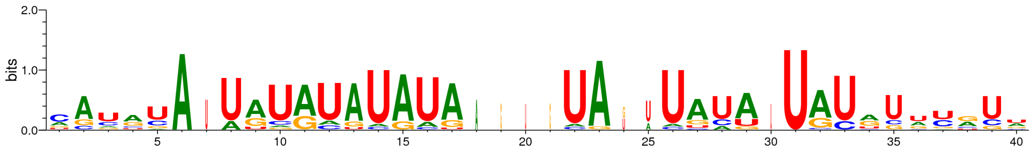

Struct.: :<<<-<<<--<<<______>>>-->>>->>>:
Consensus: AcacAcAuaUaUaUAUAgauAuACaUgUgugu

RNA motif: **m03420**

Seq. logo:

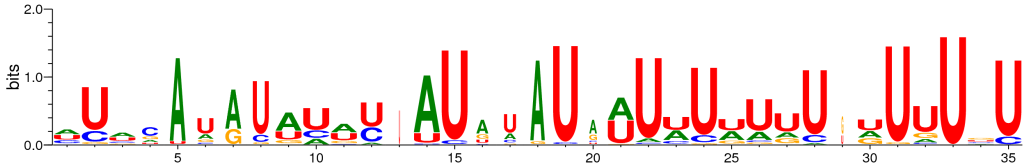

Struct.: ::::::::::::::::::::::::::::::::

Consensus: aUaCAuAUAuAUAUAUAUAUAUAUuUaUuUgU

RNA motif: **m03430**

Seq. logo:

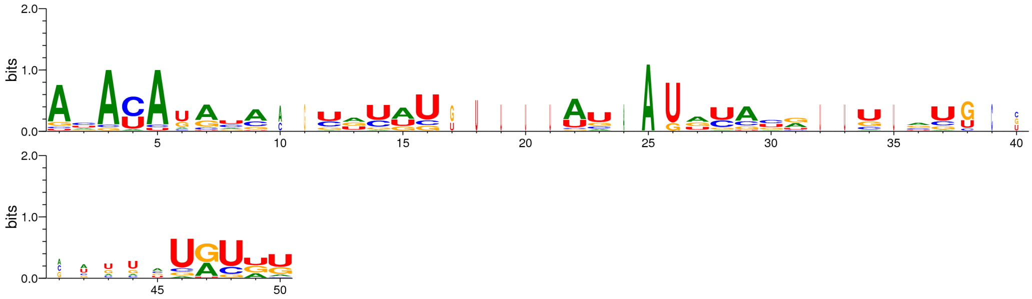

Struct.: :<<<<-<<<<<<<<____>>>>>>>>->>>>:

Consensus: ACACAuacacgcauAuAUaugcguguGUGUGu

RNA motif: **m03440**

Seq. logo:

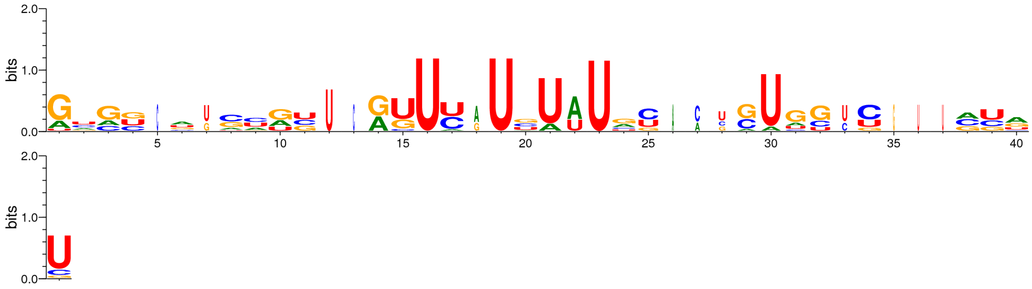

Struct.: <<<<-<<<<<<_______>>>>>>->>>>

Consensus: gcgcaCCaCGgUcUCUAUcCGuGGcgcgc

RNA motif: **m03450**

Seq. logo:

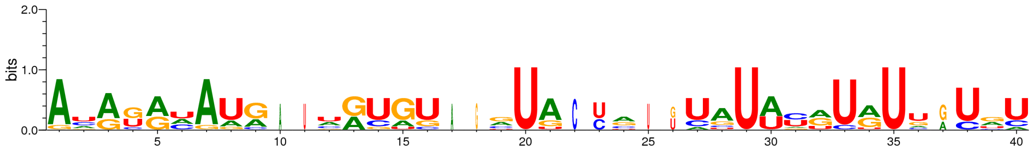

Struct.: :<<<<<<<<<<<<<___>>>>>>>>>>>>>:

Consensus: AcaggcauGuacgggUAccguaCgugucugU

RNA motif: **m04070**

Seq. logo:

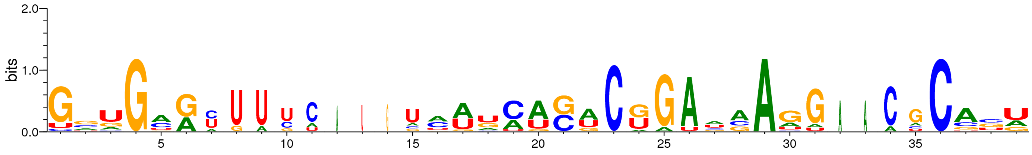

Struct.: :<<<<<______________________>>>>>:

Consensus: GcUGCGuUUuCUCuuCaCaCgGAcaAGGCGCAga

RNA motif: **m04120**

Seq. logo:

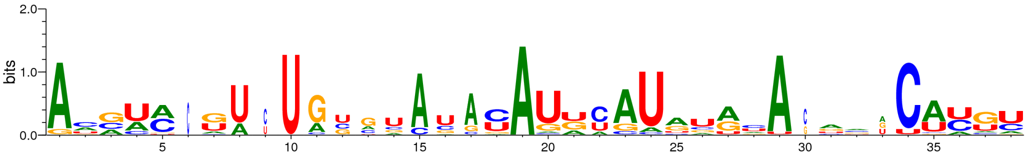

Struct.: ::::::<<<<<<__________>>>>>>::::::
Consensus: ACguCguUGCGuAUACAUgCAUaCGCAaCCaCgu

RNA motif: **m04130**

Seq. logo:

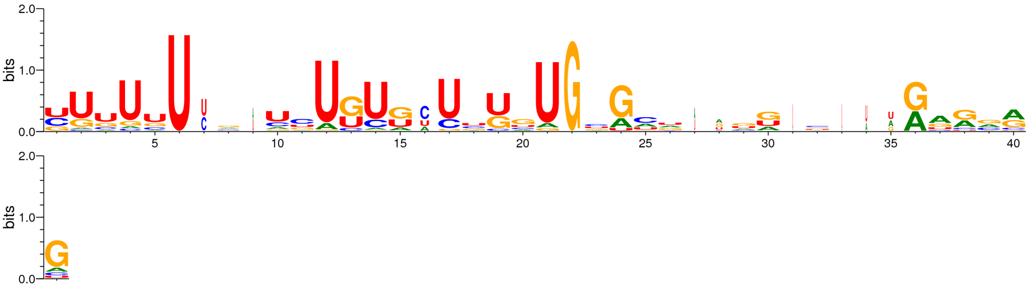

Struct.: :<<<<<<<<-<<________>>->>>>>>>>:
Consensus: CugucugccUgugUcugUGcgcaggcgggcaG

RNA motif: **m04144**

Seq. logo:

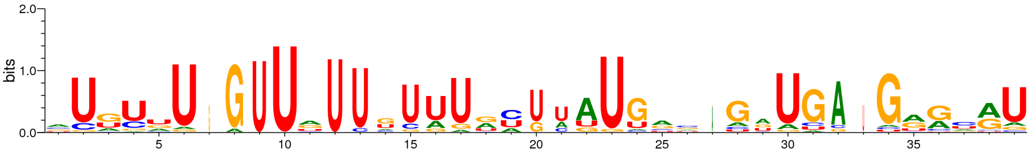

Struct.: :<<<<<-----<<<<_______>>>>---->>>>>:
Consensus: auGCuUGUUAUUGuUUaCucAUGaCGaUGAGaGCaU

RNA motif: **m04650**

Seq. logo:

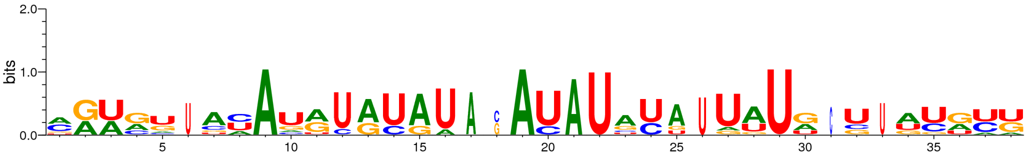

Struct.: :<<<<<<<<<<<<<____>>>>>>>>>>>>>:
Consensus: CGuguaCAuauGuaUAUAuaCauaUGuacgCg
